# Paediatric procedural sedation and analgesia in a South African emergency centre: a single-centre, descriptive study

**DOI:** 10.1186/s12245-023-00508-x

**Published:** 2023-05-15

**Authors:** Cornelle Dunn, Philip Cloete, Colleen Saunders, Katya Evans

**Affiliations:** 1 Division of Emergency Medicine, Faculty of Health Sciences, University of Cape Town, Cape Town, South Africa; 2 Groote Schuur Hospital, Observatory, Cape Town, 7701 South Africa

**Keywords:** Emergency centre, Procedural sedation and analgesia, Paediatrics, Fasting status

## Abstract

**Background:**

Procedural sedation and analgesia are considered a core competency in emergency medicine as patients present to the emergency centre on an unscheduled basis, often with complex complaints that necessitate emergent management. Previous evidence has consistently shown that procedural sedation and analgesia in the emergency centre in the paediatric population, even the very young, are safe if appropriate monitoring is performed and appropriate medications are used. The aim of the study was to describe the indications for procedural sedation and analgesia, the fasting status of paediatric patients undergoing procedural sedation and analgesia and the complications observed during procedural sedation and analgesia in the paediatric population at a single emergency centre in Cape Town, South Africa.

**Methods:**

A retrospective, descriptive study was conducted at Mitchells Plain Hospital, a district-level hospital situated in Mitchells Plain, Cape Town. All paediatric patients younger than 13 years of age who presented to the emergency centre and received procedural sedation and analgesia during the study period (December 2020–April 2021) were included in the study. Data was extracted from a standardised form, and simple descriptive statistics were used.

**Results:**

A total of 113 patients (69% male) were included: 13 infants (< 1 year of age), 47 young children (1–5 years of age) and 53 older children (5–13 years of age). There was only 1 (0.9%) complication documented, which was vomiting and did not require admission. The majority of patients received ketamine (96.5%). The standardised procedural sedation and analgesia form was completed in 49.1% of cases. Indications included burns debridement (11.5%), suturing (17.7%), fracture reduction (23.9%), lumbar punctures (31.9%) and others (15.0%). The indications for procedural sedation and analgesia varied between the different age groups. The majority of patients in this study did not have their fasting status documented (68.1%), and 18.6% were not appropriately fasted as per American Society of Anaesthesiology guidelines. Despite this, there was an extremely low rate of documented complications of 0.9%.

**Conclusion:**

The study findings are in accordance with previous international literature reporting low complication rates. Although fasting status was unknown in the majority of patients, there was an extremely low rate of documented complications and no interventions required. Safe, timely procedural sedation and analgesia with minimal pain and unnecessary suffering can become the norm in emergency medicine practice in South Africa.

**Supplementary Information:**

The online version contains supplementary material available at 10.1186/s12245-023-00508-x.

## Introduction

Emergency medicine was first introduced in the South African setting in the late 1990s. In 2003, emergency medicine was included in the list of recognised specialties [1]. Patients present to the emergency centre (EC) on an unscheduled basis, often with complex complaints that necessitate emergent management [2]. As the field of emergency medicine has developed locally and internationally, the services provided by the relevant clinicians have also evolved, and procedural sedation and analgesia (PSA) are now considered a core competency for any clinician working in the EC.

PSA is defined as the use of pharmacological agents, such as sedatives and analgesics, to alleviate anxiety, pain and fear during diagnostic and therapeutic procedures [3–5]. The South African Society for Anaesthesiologists (SASA) defines the goals of procedural sedation as reducing the patient’s fear, anxiety and distress while minimising physical discomfort and pain, thus preventing psychological trauma while maintaining control of physiological parameters to ensure patient safety [5–7]. Untreated pain can result in long-term physical, physiological and psychological effects; it is thus of utmost importance to provide relief of procedural pain and anxiety, especially in children [8, 9].

There has been an increase in research evaluating current practice, guidelines and possible complications associated with the provision of PSA in the paediatric population outside of theatre [10]. One of the recent areas of interest is the fasting status of the paediatric patient undergoing PSA and whether adhering to the standard guidelines in anaesthesia is necessary [10–13]. Multiple studies have been performed assessing the safety and efficacy of PSA in the paediatric population, all showing that PSA can be safely and effectively provided by non-anaesthesiologists in a paediatric EC [10, 14, 15]. However, most of this research has been done in the USA and Europe, with minimal research performed in the low- and middle-income countries (LMICs). There is a significant difference between anaesthetic services required and the availability of such services in many resource-limited settings, including sub-Saharan Africa. This limitation in anaesthesia availability is even more pronounced for children than for adults, as provider training and comfort with PSA in the young are uncommon. As LMICs have unique challenges, including higher volumes of paediatric patients, fewer resources, higher acuity and less paediatric specific centres, it is of value to do more research in these settings [6, 16]. The current study will add to the body of research, specifically in LMICs.

### Aims

The aim of this study was to describe the indications for PSA in the paediatric EC population, the fasting status of paediatric patients undergoing PSA and the complications observed during PSA in a single Western Cape emergency centre.

## Methods

### Study design

A retrospective chart review was conducted at Mitchells Plain Hospital, a district-level hospital situated in Mitchells Plain, Cape Town. The study was approved by the Human Research Ethics Committee of the University of Cape Town (HREC REF: 859/2019), and facility approval was granted by the National Health Research Database (WC_202104_029).

### Study setting and patients

Mitchells Plain is a suburb in Cape Town, South Africa, with a population of approximately 310,485 people. According to the latest census in 2011, the majority of the population lives in formal dwellings (95%) with running water and electricity [17]. Mitchells Plain Hospital is a 230-bed hospital. The hospital provides care at a district level, including emergency care, obstetrics and gynaecology, medical, surgical and paediatric care. On average, approximately 3926 patients attend the EC every month, 816 being paediatric [18]. All patients (younger than 13 years of age) who presented to the emergency centre of Mitchells Plain Hospital and received PSA during the study period (December 2020–Aril 2021) were included in the study.

### Data collection and management

A standardised form for collecting clinical information on paediatric PSA was designed by the EC management team and implemented in February 2020 as part of clinical quality assurance procedures at the hospital (Additional file 1). This form was based on a similar form in use successfully at another local district hospital, as well as international documentation guidelines including the Royal College of Emergency Medicine, New South Wales Government in Australia, and Mount Sinai School of Medicine PSA form and forms published by SASA for use in paediatric PSA [19–23]. The PSA form has a barcode and is included in the standard documentation captured on the electronic content management (ECM) system. It is completed by medical staff for all paediatric patients undergoing PSA.

**Fig. 1:**
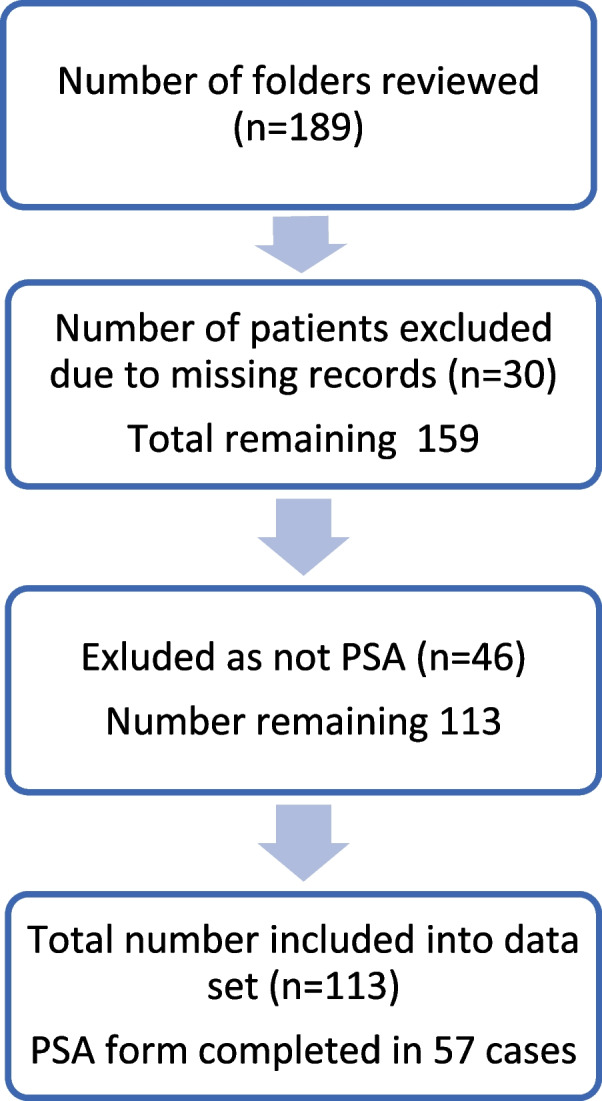
Flowchart of patient selection

Paediatric patients who received PSA were identified by reviewing the scheduled drug register in the paediatric EC. All scheduled drugs are recorded with the patient name as well as folder number. Once eligible patients were identified, their medical records were reviewed. Those who received scheduled drugs for purposes other than PSA were excluded. Information was retrospectively abstracted from the standardised PSA forms and electronic patient care records for all paediatric patients who underwent PSA in the EC between December 2020 and April 2021, and data was subsequently deidentified prior to analysis. In cases where the standardised PSA form was missing, not utilised or incomplete, missing information was extracted from the medical records, including doctor notes, progress reports by nursing staff and referral letters. PSA forms that were missing information on the fasting state were included in order to provide information on secondary outcomes. Patients were excluded from the study if no notes or records could be traced relating to the procedure (Fig. 1). Patients were considered to be appropriately fasted if they met the American Society of Anaesthesiology (ASA) guidelines which require the patient to be nil per os (NPO) for 6 h for solids, 4 h for breast milk and 2 h for any clear fluids [24].

### Statistical analysis

Data was captured into a password-protected, Microsoft Excel spreadsheet. Identifying information from the data set was removed before analysis. Descriptive statistics were used to describe the demographics of the study sample, indications for PSA, drugs and dosages administered and complications associated with PSA. The patient population was divided into three groups: infants (< 1 year of age), younger child (1–5 years of age) and older child (5–12 years of age). Where applicable, categorical data was compared using the chi-square test. The fasting state of the patients was also assessed within the context of various age groups. As this was the primary objective of the study, if the fasting state of the patient was unknown, this was specifically noted and described. Data analysis was performed using Microsoft Excel 365 ProPlus.

## Results

### Demographics

A total of 189 patient folders were screened, of which 73 were excluded (incorrect folder number (*n* = 11), drugs administered were not for the purpose of PSA (*n* = 46) and no clinical records found (*n* = 19)). Therefore, 113 patients in three age categories were included in the study (Table 1): 13 infants (< 1 year of age), 47 young children (1–5 years of age) and 53 older children (5–12 years of age). The majority of the patients were male (*n* = 78, 69.0%) with a male predominance more apparent in the older children (79.2%) than in the infants (46.2%).

**Table 1:** Demographic and clinical characteristics of patients

	***All*** ***N* = *113***	***Infants*** ***N* = *13***	***Young children*** ***N* = *47***	***Older children*** ***N* = *53***
**Sex*,**** n (%)*				
*Male*	78 (69.0)	6 (46.2)	30 (63.8)	42 (79.2)
***Weight (*kg; *mean* ± *SD)***				
	17.3 ± 7.6	8.1 ± 2.8	13.1 ± 3.6	23.3 ± 6.3
***Performed after hours n (%)***	85 (75.2)	10 (76.9)	37 (78.7)	38 (71.7)
***Indications n (%)***				
*Burns debridement*	13 (11.5)	3 (23.1)	8 (17.0)	2 (3.8)
*Fracture reduction*	27 (23.9)	1 (7.7)	3 (6.4)	23 (43.4)
*Suturing*	20 (17.7)	1 (7.7)	10 (21.3)	9 (17.0)
*Lumbar puncture*	36 (31.9)	7 (53.8)	22 (46.8)	7 (13.2)
*Other*^*a*^	17 (15.0)	1 (7.7)	4 (8.5)	12 (22.6)
***Disposition n (%)***				
*Admit*	23 (20.4)	4 (30.8)	15 (31.9)	4 (7.5)
*Discharge*	63 (55.8)	5 (38.5)	21 (44.7)	37 (69.8)
*Refer to RCWCH*^*b*^	23 (20.4)	3 (23.1)	9 (19.1)	11 (20.8)
*Not documented*	4 (3.5)	1 (7.7)	2 (4.3)	1 (1.9)

^a^Removal of foreign body, catheterization, intercostal drain insertion, CT scan, incision and drainage of abscess, examination, reduction of paraphimosis

^b^RCWMCH, Red Cross War Memorial Children’s Hospital

PSA was most commonly indicated for lumbar puncture (31.9%) and fracture reduction (23.9%); however, indications varied between the different age groups with burns debridement being the highest in the infant population (23.1%) and lowest in the older child group (3.8%) (Table 1). Fracture reduction was highest in the older child group (43.4%) and lowest in the younger child group (6.4%). LPs had highest incidence in the infant group followed by the younger child group (53.8% and 46.8%). The younger groups were also more likely to need admission.

Of the 113 patients, only 57 (50.4%) patient folders contained a completed PSA form. Most of the patients in this study received ketamine (109). The drug of choice was not documented in four of the patients. Drug choice, and dose (mg/kg), did not vary significantly across the different age groups. Most of the patients received PSA after hours (between 16:00 and 08:00). Findings are summarised in Table 2.

**Table 2:** Fasting status, complications, drugs used and providers

	***All*** ***N* = *113***	***Infant*** ***N* = *13***	***Young child*** ***N* = *47***	***Older child*** ***N* = *53***
***Appropriately fasted,**** n (%)*				
*Yes*	15 (13.2)	1 (7.7)	6 (12.8)	8 (15.1)
*No*	21 (18.6)	2 (15.4)	10 (21.3)	9 (17.0)
*Unknown*	77 (68.1)	10 (76.9)	31 (66.0)	36 (67.9)
***Complications, n (%)***				
*Vomiting*	1 (0.9)	0	0	1 (1.9)
*Nil documented*	107 (94.7)	13 (100)	44 ( 93.6)	50 (94.3)
*Unknown*	5 (4.4)	0	3 (6.4)	2 (3.8)
***Drug used n (%)***				
*Ketamine*	109 (96.5)	13 (100)	44 (93.6)	52 (8.1)
*Unknown*	4 (3.5)	0	3 (6.4)	1 (1.9)
***Number of doses (median/IQR)***	1 (1–1)	1 (1–1)	1 (1–1)	1 (1–1)
***Range of doses***	1–3	1–2	1–2	1–3
***Dosage (mg/kg) — mean and SD***	1.48 ± 0.89	1.55 ± 0.96	1.60 ± 1.05	1.36 ± 0.69
***Most senior provider n (%)***				
*Intern*	13 (11.5)	2 (15.4)	7 (14.9)	4 (7.5)
*COSMO*^*a*^	43 (38.1)	3 (23.1)	16 (34.0)	24 (45.3)
*MO*^*b*^	12 (10.6)	2 (15.4)	4 (8.5)	6 (11.3)
*Registrar*	15 (13.3)	4 (30.8)	5 (10.6)	6 (11.3)
*Unknown*	30 (26.5)	2 (15.4)	15 (31.9)	13 (24.5)

^a^Community service medical officer

^b^Medical officer

The majority of patients in this study were not appropriately fasted, as per ASA guidelines, or the fasting status was unknown. Despite this, there was an extremely low rate of documented complications of 0.9%. The drug of choice was ketamine with a mean dosage of 1.48 ± 0.89 mg/kg.

## Discussion

Procedural sedation and analgesia form part of everyday management in the EC. Published literature consistently shows that PSA in the emergency centre in the paediatric population is safe if appropriate monitoring is performed and appropriate medications are used, even in the very young [10, 15, 25]. The most important finding in this study is the low rate of complications following PSA within the paediatric population in the EC. Out of the 113 patients, only one patient had a documented complication being vomiting. There was no incidence of laryngospasm and no aspiration, and the patient did not require admission following this complication. This finding is consistent with previously published findings. Misra et al. [25] assessed the safety of PSA in the very young (patients less than 2 years old) and concluded that children under the age of 2 years can safely undergo PSA in the EC without increased risk of adverse events [25]. In the current study, the patient with a documented complication was an older child, and no complications were observed in the very young.

In 2016, Woo et al. [25] conducted a study in Korea to assess patient factors associated with adverse events. The basis for their study was that ketamine is commonly used in the ED for paediatric PSA, although patient factors associated with adverse events are poorly described. They found no significant association between the duration of fasting and adverse events (*P* = 0.073) or between food type and adverse events (*P* = 0.734). However, administration route and dosage were associated with adverse events in children sedated with ketamine in the EC [26].

Beach et al. [13] evaluated the fasting status in paediatric patients undergoing PSA and whether adhering to standard NPO guidelines, as set out by the ASA, influenced the complication rate in these patients. The authors showed that aspiration is uncommon, and that fasting status for both liquids and solids is not an independent predictor of major complications nor aspiration during PSA [13].

In 2017, Bhatt, Johnson, and Chan [5] described 6295 paediatric patients who underwent PSA in the EC for painful procedures. This is one of the largest studies to date and was conducted in six emergency departments across Canada. They found adverse events reported in 11.7% of patients with the most common adverse events being vomiting and desaturation. Serious adverse events occurred in only 1.1% of patients and 1.4% required intervention with positive pressure ventilation being the only significant intervention. There were no unplanned admissions to hospital due to the adverse events, and sedation was successful in 95% of cases. They found that the incidence of adverse events varied significantly with the choice of sedative; the lowest incidence was with ketamine single agent as compared to the combination of ketamine and fentanyl or ketamine and propofol. In this large, multicentre cohort study, ED procedural sedation was performed safely in the paediatric population with a low overall incidence of adverse events.

In 2018, Green, Leroy, Roback, Irwin, Andolfatto, and Babl published an editorial calling for a cautious but progressive application of more liberal fasting guidelines in EC PSA [12]. A prominent mention in their editorial was that aspiration in the healthy child is very rare, and they suggest that the risk of pulmonary aspiration in healthy children receiving PSA is functionally negligible. The authors concluded by stating that the time for fasting reform is due [12]. The multidisciplinary International Committee for the Advancement of Procedural Sedation was established in 2019 and subsequently developed the first fasting and aspiration prevention recommendations specific to procedural sedation [22], as well as guidelines pertaining to PSA in the EC [22]. SASA first published guidelines for PSA in the paediatric population in 2010 and updated these guidelines in 2016 and 2021 [6]. The guidelines for fasting in the SASA guidelines have also been amended in the latest version and now recommend standard anaesthesia fasting guidelines for advanced sedation, but where simple sedation is planned, no fasting is required [6].

Although many studies published within this field, few studies could be found specifically describing the relationship between fasting status and complications in paediatric ECs in LMICs [2, 3, 5, 13, 15, 25]. Low-resource settings have many challenges that serve as barriers when it comes to health care. Some of the most precious resources are time, beds, space, and the specialists available. It is therefore essential to provide PSA safely to the paediatric patient outside of theatre [1, 2, 4]. Applying evidence-based medicine and newly developed guidelines can provide PSA safely with minimal delays and thus save time, money, and other resources [27, 28].

Similar to other resource-limited settings, Tanzania has also done research into the implementation of ketamine PSA by the emergency centre, assessing patient safety, adverse events and patient and provider satisfaction [29]. Fasting requirements in the EC followed the latest emergency care guidelines recommending that procedural sedation not be delayed for adults or children based on fasting times. Many of their patients had oral intake within 4 h of ketamine administration. There were no cases of vomiting or pulmonary aspiration during the procedures. A total of 12% of the patients reported nausea and vomiting post-procedure. The indications for and experience with ketamine for procedural sedation in their EC in Tanzania were similar to findings from high-resource settings. There were no serious adverse events attributable to ketamine despite a high-acuity and the frequency of non-fasted patients in the sample [29].

Fasting status in our study was only documented in a third of the patients (*n* = 36, 31.9%), and only 15 (13.3%) of those patients were appropriately fasted according to the ASA guidelines. Yet, only one patient in this study had a documented complication, vomiting. Our findings therefore support the international published literature indicating that PSA in the paediatic patient did not show an increase in interventions nor complications, despite the fasting status, in previously healthy children [12–14, 22, 30].

Medical records are a crucial part of a patient’s journey. These serve as a permanent record of the patient’s illness and medical care, enabling clinicians to make informed clinical decisions [31]. As healthcare professionals, we need to strive for the highest standards of clinical documentation [31]. It allows for the scientific evaluation of patient profiles, analysing treatment results and facilitate planning of treatment protocols. Medical records are also crucially important when one has to address issues of alleged medical negligence [32].

Various centres have developed their own standardised forms to be completed when performing PSA [6]. During the data collection process, it was noted how the documentation regarding PSA in the EC varies. In the current study, the form was only used in 49.1% of cases despite its inclusion in the standard documentation captured on the ECM system and an expectation that it is completed by medical staff for all paediatric patients undergoing PSA. Documentation was found to be incomplete, information inconsistent and very succinct. Both doctors and nursing notes had to be reviewed in order to find detailed information about the procedure. Documentation of the complications was clear, concise with a clear disposition and follow-up plan, acknowledging that in this study, there was only 1. Despite the lack of documented complications, we would be remiss to not take into account the lack of documentation in a large proportion of our study population which could have led to omitting undocumented complications. There are multiple possible reasons for this, including regular locum doctors, rotation of junior doctors not being aware of the form and the impact of the COVID-19 pandemic. The form was rolled out just before the first wave of COVID-19, and as the focus in health care shifted to managing the COVID-19 pandemic, it is reasonable to assume that this has also led to doctors not using the form or implementation not being as successful as expected.

Documentation remains a challenge, with medical records often being handwritten and liable to misinterpretation due to illegibility and misplacement. This can affect the patient’s medical care and has medicolegal implications [31]. In high-resource settings, electronic medical recordkeeping has been utilised successfully for several years. The paediatric department at Queen’s Hospital Burton successfully implemented a fully integrated electronic health record (EHR) system. This EHR improved the paediatric clinical documentation standards to 100% in each domain compared with pre-implementation standards [31].

Similarly, the Emergency Medicine Department at Muhimbili National Hospital (MNH) in Tanzania implemented the first electronic medical record (EMR) tailored to the emergency centre (EC) in Tanzania in 2015 [33]. They utilised an Emergency Department Information System (EDIS), which has improved access to data and EC reports and produced research projects [33].

In the current study, we observed that the majority of PSA was provided by community service medical officers (COSMO) and medical officers (MO). Wenzel-Smith and Schweitzer [8] assessed the safety of PSA (in all ages) provided by MOs in a district hospital in the Western Cape using a retrospective chart review [16]. Their single-centre study aimed to show that PSA can be provided safely and effectively by MOs with no formal training in anaesthesia. They found low complication rates with higher complication rates in the older population (median age 40 years) with more comorbidities and medication use compared to the young (median age of 22 years) [16]. The authors speculated that MOs were more reluctant to use higher doses of medication in the paediatric population and used mostly single agents rather than a combination [16].

In 2016, Swartz et al. [33] published their research done in rural Western Kenya. They developed a pilot programme — every second matter for mothers and babies — ketamine (ESM-Ketamine) [34, 35]. Their study included 90 children below 18 years who underwent PSA with ketamine for emergent procedures when no anaesthetist was available. The mean age was 10.6 years. They found that 17% of patients experienced minor adverse events, including hallucinations, hypersalivation and desaturation to SaO2 below 92% for less than 30 s. All adverse events self-aborted, and none of the patients required any intervention with no reported serious adverse events. The use of the ESM-Ketamine care package allowed procedures to be performed timeously without pain, decreasing delays and suffering [34]. These findings are consistent with our results.

The Red Cross War Memorial Children`s Hospital (RCWMCH) is the largest paediatric hospital in Africa and is situated in Cape Town. The hospital identified the need for an out-of-theatre sedation service due to the increased need for PSA [4]. They did an observational study in 2019 with the primary aim of defining the number of cases of PSA performed outside of operating theatre. They excluded any PSA performed in the trauma unit and ICU seeing as these units will not benefit from the out-of-theatre sedation service. They reviewed 288 sedations; the overall complication rate was low and in line with international literature. These complications included the following: airway obstruction (4.9%), desaturation (4.2%) (defined as saturation < 90% for > 60 s), laryngospasm (0.3%) and nausea and vomiting (2.4%) [4].

The evidence from these internationally conducted studies highlights the safety of PSA provision within the EC by MOs without formal anaesthetic training [16]. The findings from our study are consistent with this literature, safely supporting less stringent fasting guidelines for PSA in the EC. The use of PSA has been associated with a reduction in healthcare cost and hospital length of stay which will potentially reduce the financial burden on health care across Africa and other resource-constrained environments [1].

### Strengths and limitations

The validity and reliability of the study findings were enhanced by using a single-trained investigator to perform the data collection. All medical records of study patients were reviewed in order to limit the missing data. However, the study still had several limitations. Only data documented by practitioners could be collected. Only 49.1% of patients had the standardised form completed. As the schedule drug book was used as a reference to trace patients receiving PSA, we relied on the information recorded there. Names, as well as folder numbers, were often incorrect, leading to exclusion of patients. There is also the chance that some patients might have received PSA without the drugs being documented within the scheduled drug book.

## Conclusions

Our study findings are in line with research conducted internationally. The emergency centre PSA in the paediatric populations shows low rates of complications and interventions required to manage complications, despite the non-fasted state and lack of documentation of fasting status [22]. This has practice implications for our ECs. Keeping children in the EC to meet fasting guidelines as set out by the ASA leads to prolonged stay, increased cost, increased nursing burden and increased distress to the child and parents. By incorporating the findings described from our study combined with international literature, safe, timely PSA with minimal pain and unnecessary suffering can become the norm in emergency medicine practice in SA.

### Future directions

Utilising a standardised form as part of PSA within our setting will lead to the capacity to perform larger studies. Implementing this into practice, educating all practitioners regarding it and then performing audits could lead to a larger study population and allow more definitive results. This could lead to potential provincial or national implementation of the form or similar forms across all ECs and will facilitate a measurable standard of care in emergency PSA in the paediatric population.

## Supplementary Information

**Additional file 1.** Procedural Sedation and Analgesia – Paediatric patients. 

## Data Availability

The dataset used during the current study is available from the corresponding author on reasonable request and pending further ethical and facility approval.
