# Supplementary material for: Paediatric procedural sedation and analgesia in a South African emergency centre: a single-centre, descriptive study
Source: Int J Emerg Med. 2023 May 15;16:37. doi: 10.1186/s12245-023-00508-x (PMC10183245; doi:10.1186/s12245-023-00508-x)
Supplement: Supplementary file 1 — Additional file 1. Procedural Sedation and Analgesia – Paediatric patients. [file 12245_2023_508_MOESM1_ESM.docx]

Additional file 1

Procedural Sedation and Analgesia – Paediatric patients

| **DATE:** |
| --- |
|  |
| **WEIGHT:** |

Patient sticker

Name:

Folder nr:

DOB:

**Fasting Status:** **Team members involved in Sedation**

| **LAST ORAL INTAKE** | **TIME** |  | **Name & Surname** |  | *(circle the correct answer)* |
| --- | --- | --- | --- | --- | --- |
| Clear fluids | H |  |  | Doctor | Consultant/Reg/MO/COSMO/Intern |
| Breastmilk | H |  |  | Doctor | Consultant/Reg/MO/COSMO/Intern |
| Formula / solids | H |  |  | Nurse | CNP/PN/EN/ENA |

**Procedure: 0 Consent Done 0 EMLA used yes/no**

| Lumbar Puncture |  |  | **Procedure Notes:** |
| --- | --- | --- | --- |
| Fracture reduction |  |  |  |
| Suturing |  |  |  |
| Removal ear/nose foreign body |  |  |  |
| Examination |  |  |  |
| Other:___________ |  |  |  |
|  |  |  |  |

| Drug | Dose #1 | Time | Sign | Dose #2 | Time | Sign |
| --- | --- | --- | --- | --- | --- | --- |
|  |  | H |  |  | H |  |
|  |  | H |  |  | H |  |
|  |  | H |  |  | H |  |

**Monitoring**: 0 SpO2 0 ETCO2 0 ECG 0 BP

|  | Pre-procedure | 5 Min | 10 Min | 15 Min | 20Min | 25Min | 30 Min | Final |  |
| --- | --- | --- | --- | --- | --- | --- | --- | --- | --- |
| FiO2 |  |  |  |  |  |  |  |  |  |
| BP |  |  |  |  |  |  |  |  |  |
| Pulse |  |  |  |  |  |  |  |  |  |
| SaO2 |  |  |  |  |  |  |  |  |  |
| RR |  |  |  |  |  |  |  |  |  |

Time fully awake:

Monitored by whom during recovery (e.g. mom/nurse/doctor)

Complications:

| Vomiting |  |
| --- | --- |
| Apnoea |  |
| Cardiac instability |  |
| Laryngospasm |  |
| None |  |

Management: please provide brief summary

Note-keepers name & signature

Disposition:

| Discharge |  | Admission |  | Other |  |
| --- | --- | --- | --- | --- | --- |

Commonly used drugs:

| Drug | IMI dose | IV dose | Oral dose |
| --- | --- | --- | --- |
| Ketamine | 4mg/kg | 0.5-1mg/kg | 4-5mg/kg |
| Fentanyl |  | 1ug/kg |  |
| Midazolam |  | 0.05-0.1mg/kg |  |
| Propofol |  | 0.5-1mg/kg |  |
